# Microfluidic Technology for the Isolation and Analysis of Exosomes

**DOI:** 10.3390/mi13101571

**Published:** 2022-09-22

**Authors:** Yusong Wu, Yuqing Wang, Yanjun Lu, Xiaomei Luo, Yinghong Huang, Ting Xie, Christian Pilarsky, Yuanye Dang, Jianye Zhang

**Affiliations:** 1 Guangzhou Municipal and Guangdong Provincial Key Laboratory of Molecular Target & Clinical Pharmacology, NMPA and State Key Laboratory of Respiratory Disease, School of Pharmaceutical Sciences and the Fifth Affiliated Hospital, Guangzhou Medical University, Guangzhou 511436, China; 2 Guangdong Provincial Key Laboratory of Micro/Nano Optomechatronics Engineering, College of Mechatronics and Control Engineering, Shenzhen University, Shenzhen 518060, China; 3 Department of Surgery, Friedrich-Alexander University of Erlangen-Nuremberg (FAU), University Hospital of Erlangen, 91054 Erlangen, Germany

**Keywords:** exosomes, extra-cellular vesicle, microfluidics, isolation, detection

## Abstract

Exosomes are lipid-bilayer enclosed vesicles with diameters of 30–150 nm, which play a pivotal role in cell communication by transporting their cargoes such as proteins, lipids, and genetic materials. In recent years, exosomes have been under intense investigation, as they show great promise in numerous areas, especially as bio-markers in liquid biopsies. However, due to the high heterogeneity and the nano size of exosomes, the separation of exosomes is not easy. This review will deliver an outline of the conventional methods and the microfluidic-based technologies for exosome separation. Particular attention is devoted to microfluidic devices, highlighting the efficiency of exosome isolation by these methods. Additionally, this review will introduce advances made in the integrated microfluidics technologies that enable the separation and analysis of exosomes.

## 1. Introduction

**Figure 1:**
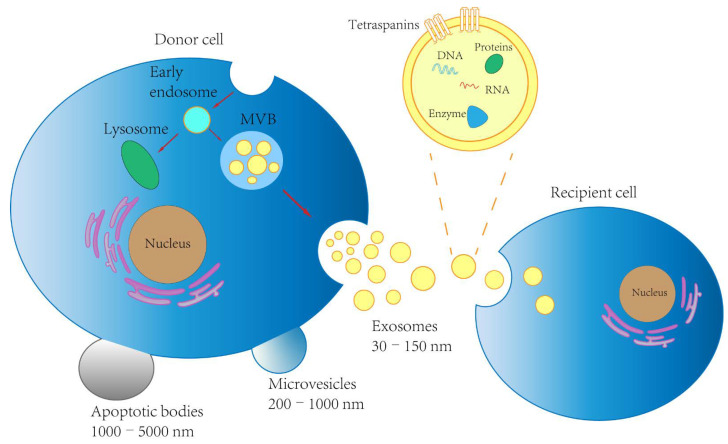
The biogenesis and content of exosomes. First, the plasma membrane depresses inward to form an early endosome, then multivesicular bodies (MVBs) are formed by the inward budding of endosomal membranes. Finally, exosomes are released by the fusion of MVBs with the plasma membrane.

Exosomes were first identified in sheep reticulocytes in 1983 and named “exosome” by Johnstone in 1987 [1]. Although they were initially considered to be the waste exuded by cells, they have now become a key field for research, as various pieces of research have shown that exosomes play a pivotal role in cell communication [1,2,3]. Exosomes are one subset of extra-cellular vesicles (EVs), which are mainly divided into three subtypes, cell microvesicles (MVs), apoptotic vesicles, and exosomes, based on their differences in size and origin [4,5]. Exosomes can provide a variety of bioactive molecules to recipient cells and reflect the information of their cell origin, as shown in Figure 1 [6,7,8]. There are some studies that have shown that exosomes secreted by various of cancers express different levels of proteins. For example, Jakobsen et al. found that lung-cancer-cell-derived exosomes can highly express CD91 and epidermal growth factor receptor (EGFR) [9]. Melo et al. reported glypican-1 (GPC1) specifically enriched in exosomes from the patients with pancreatic cancer [10].

**Figure 2:**
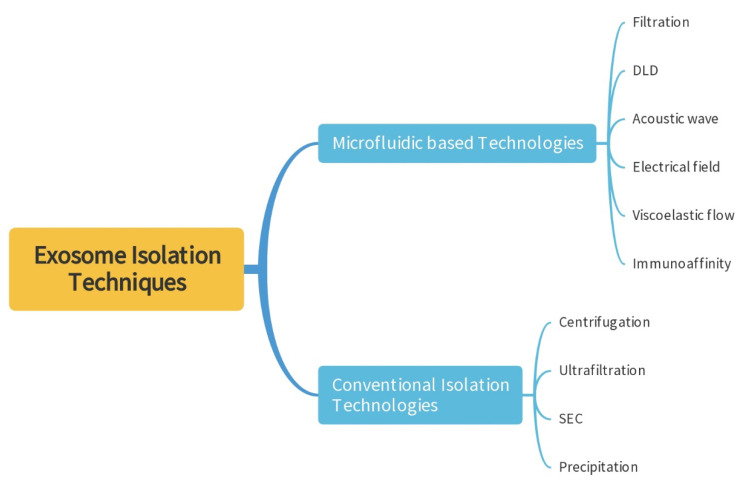
Schematic summary of techniques for exosome separation.

Exosomes are lipid-bilayer enclosed vesicles with a diameter of 30–150 nm, which can be released by most cells and be found in several biofluid types such as blood, urine, bile, saliva, and breast milk [2,11]. Meanwhile, exosomes have the following characteristics. First, exosomes are abundant in biological body fluids. Second, exosomes are secreted by most living cells. Third, exosomes are relatively stable because of their lipid bilayer [2,12]. Therefore, exosomes are advantageous in liquid biopsies based on the above characteristics [13]. However, due to the high heterogeneity and the nano size of exosomes, the relative research and application of exosomes has been limited [14]. Although there are many methods for the isolation and detection of exosomes, most of these methods require expensive instruments and reagents and are very time consuming [15,16]. This review will deliver an outline of the conventional methods and the microfluidic-based technologies for exosomes separation (Figure 2), along with their strengths and weaknesses. Meanwhile, most reviews pay more attention to the technology of microfluidic, not focus on the efficiency of exosome isolation. In order to evaluate the methods objectively, we summarized the yield and purity of the exosomes isolated from each method. Further, with the development of microfluidic technology in recent years, several integrated microfluidic technologies have been developed for the separation and analysis of exosomes.

## 2. Conventional Exosome Isolation Methods

In this section, several conventional exosome-separation methods will be introduced, including their working principles, advantages, and disadvantages (Table 1).

### 2.1. Ultracentrifugation (UC)

Ultracentrifugation is the most common method for the separation of exosomes and is regarded as the gold standard [17,18]. The method is based on the differences in the size and density between exosomes and other components. It can be classified into two types: differential ultracentrifugation and density gradient ultracentrifugation. For differential ultracentrifugation, the samples were first centrifuged at low speed (300× *g*) to remove cellular debris, then the large vesicles, such as apoptotic bodies and microvesicles, were removed under a higher speed centrifuge (<20,000× *g*), and finally the exosomes were precipitated under the action of high centrifugal force (more than 100,000× *g*) [1]. Density gradient centrifugation is a better separation technology based on ultracentrifugation [19]. Compared to differential ultracentrifugation, density gradient centrifugation uses a minimum of two solutions with different densities. After centrifugation, particles of different densities can be separated into similar density layers [20]. Sucrose and iodoxanol are the most widely used solutions to produce continuous gradients [21,22]. However, although it has a higher separation purity of exosomes than ultracentrifugation, density gradient centrifugation is a more time-consuming method [23]. In addition, the structure and quality of the collected exosomes may be affected by the repeated centrifugation and excessive centrifugal force.

### 2.2. Ultrafiltration (UF)

Ultrafiltration is a simple separation method to separate different sized particles by using membrane filters with appropriate pore sizes. Based on the fact that the size of an exosome is in the range of 30–150 nm, nanomembranes with different molecular weight cut-off (MWCO) are used to isolate exosomes from other particles [24]. Unfortunately, the clogging of the filtering membrane can reduce the exosome isolation efficiency and the lifetime of membranes [25]. There are two main types of ultrafiltration methods. The first is dead-end filtration, where fluid passes perpendicularly through the membrane, which will result in the rapid formation of filter cake and thus reduce the separation efficiency. This method is rarely used alone. The second is tangential flow filtration (TFF), where the fluid flows tangentially through the surface to avoid cake formation [26,27]. In addition, sequential ultrafiltration is another commonly used method. This approach is divided into three steps of dead-end filtration, tangential-flow filtration, and track-etched membrane filtration [28]. First, dead-end filtration is used to get rid of extra-cellular vesicles with a diameter greater than 100 nm. Subsequently, TFF is used to remove free proteins and other small particles. Finally, microvesicles are removed and exosomes can be collected. Ultrafiltration is usually applied to isolate exosomes from large amounts of liquid such as cell culture medium, as it does not require expensive equipment and complex procedures [29].

### 2.3. Size-Exclusion Chromatography (SEC)

Size-exclusion chromatography is a separation method that avoids exosome damage as much as possible [30,31]. When the liquid sample passes through the column consisting of porous beads, particles of different sizes have different flow rates; smaller particles such as exosomes can enter pores and the elution speed is slow, while larger components that cannot enter the pores are eluted earlier from the column [32]. This method allows exosomes to be isolated with minimal external forces, thereby reducing the impact on exosomes and maintaining the biological activities of the exosomes [31,33]. Importantly, the exosomes isolated via this method have a high level of purity, which is conducive to basic research and its clinical application [31]. In addition, this method has other merits, including requiring low-sample volumes, the exosomes are easy to collect, and it is not time or labor intensive [34,35].

### 2.4. Polymer-Based Precipitation

Polymer-based precipitation uses hydrophilic polymers such as polyethylene glycol (PEG) to combine the water molecules surrounding the exosomes to form a hydrophobic environment, which reduces the solubility of the exosomes and facilitates their precipitation [36,37]. The sample is then centrifuged at a low speed to obtain EVs [38]. Compared with the ultracentrifugation, this method can provide higher yields as well as avoid excessive centrifugation, which can cause exosome damage [39]. A variety of commercial kits used in polymer-based precipitation methods have been used to extract EVs. Tian et al. compared the performance of popular commercial kits with EV preparation by UC and found that the EVs extracted from the kits produced a higher yield but of a much lower purity [40].

## 3. Microfluidic-Based Technology for Exosome Isolation

Microfluidics is the science and technology of systems that manipulate small amounts of fluids within microscale channels [41]. Microfluidics not only have the advantage of reducing the consumption of samples and reagents and providing higher resolution and sensitivity, but they also significantly reduce the analysis time and greatly improve the analysis efficiency [42,43]. Due to the vast amounts of progress in microfabrication technology through recent years, microfluidic technology has become a promising method for exosome isolation [44]. Currently, microfluidic-based exosome-separation technologies can be classified into two types: (I) physical property-based microfluidic for exosome separation, (II) immunoaffinity-based microfluidic for exosome separation. In order to evaluate the methods, the yield and purity of each method were summarized in Table 2.

### 3.1. Physical Property-Based Microfluidics

#### 3.1.1. Exosome Isolation by Filtration

**Figure 3:**
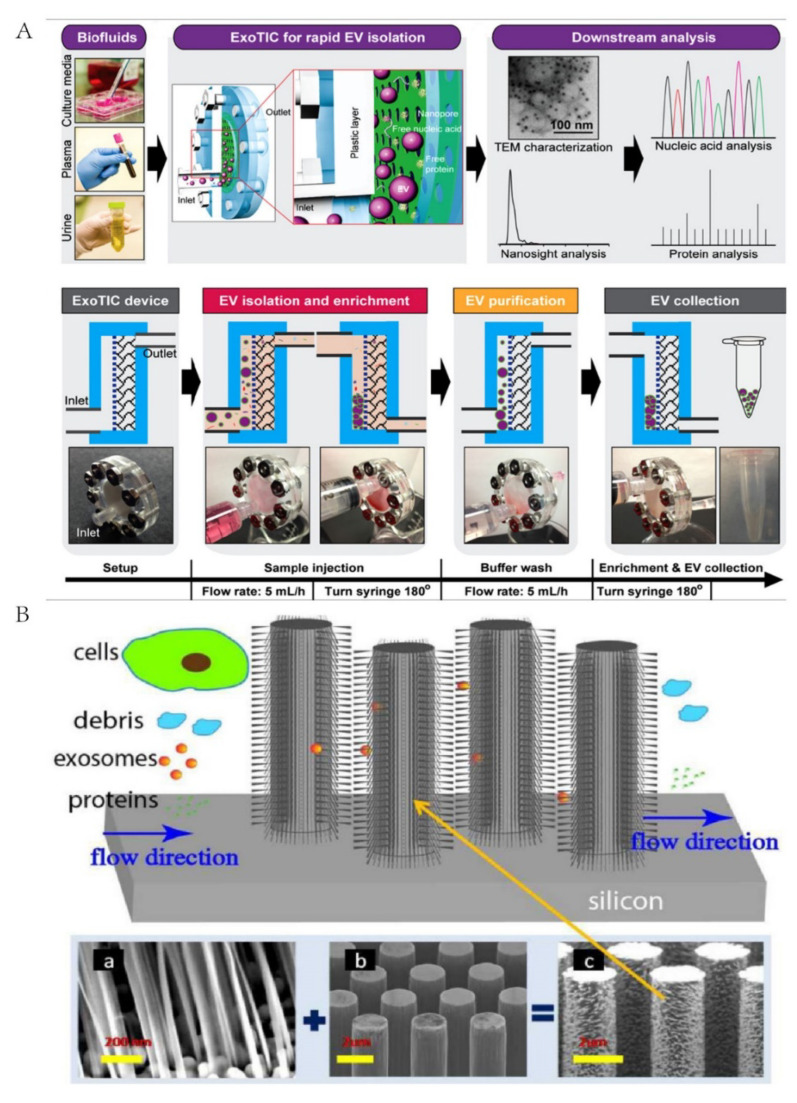
Microfluidic technology for exosome isolation based on filtration. (**A**) Schematic of ExoTIC device used by Liu et al. Reprinted with permission [45]. (**B**) Schematic of ciliated micropillar nanowires coated with nanowires. Reprinted with permission [46].

The filtration method based on microfluidic chips is a relatively simple method, which pays more attention to the design of the chip, including nanomembranes or nanowires [45,46,54,55,56,57]. Based on the size of the exosomes, nanomembranes and nanowires allow particles smaller than the pore size to enter while preventing larger particles from entering. Davies RT et al. applied a microfluidic system using porous polymer monoliths (PPMs) to isolate vesicles from whole blood [54]. The DC electrophoresis was employed as a driving force to push particles through the filter and avoid clogging. With this configuration, exosomes can be obtained from a 240 μL sample with a 2 μm min^−1^ flow rate. In order to prevent the damage to exosomes from chemical and biological reactions, Cho et al. proposed a separation system completely dependent on physical interactions [55]. Particles with different surface charges will move towards the anode or cathode under electrophoretic conditions. Among them, exosomes and most proteins have a negative surface charge, so they will move towards the anode and a nanomembrane with 30 nm pores is used to preclude the passage of the exosomes. However, as exosomes accumulate on the membrane, if the membrane cannot be cleaned in time, the separation efficiency may be reduced. Additionally, the attached exosomes, when washed and collected by PBS, may carry small proteins. Liu et al. designed a size-based isolation tool called Exosome Total Isolation Chip (abbreviated as ExoTIC) [45]. ExoTIC is a modular platform that can separate exosomes from various samples, including urine, plasma, and cell culture media. ExoTIC enriched and purified EVs using a filter, which can wash out the free proteins and nucleic acids (Figure 3A). Compared with UC, the chip can isolate exosomes from small volumes of blood (10–100 μL) with a yield 4 to 1000 times higher in <3 h. Obviously, ExoTIC is an ideal clinical device for downstream point-of-care testing (POCT).

Another novel design is nanowires; Wang et al. fabricated a microfluidic device composed of ciliated micropillars, which was formed into porous silicon nanowires [46]. The ciliated micropillars can preferentially trap exosomes and be dissolved in the PBS buffer (Figure 3B). Further, Chen et al. presented a three-dimensional PDMS scaffold chip device wrapped by ZnO nanowires [57]. The ZnO nanowire array provides a large surface area for antibody fixation and generates a size exclusion-like effect for capturing exosomes. However, despite the novelty of these designs, their structural complexity may limit their application.

#### 3.1.2. Deterministic Lateral Displacement

**Figure 4:**
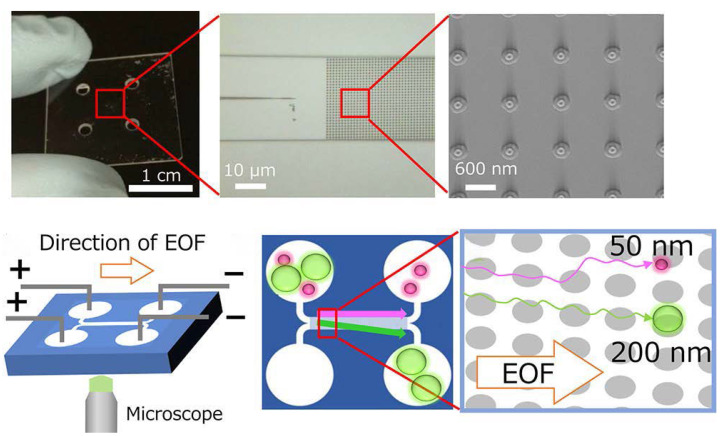
Schematic of a microfluidic device for exosome isolation based on DLD. Reprinted with permission [60].

Deterministic lateral displacement (DLD) is a size-based cell-classification technique, which is composed of a set of ordered obstacle arrays (microcolumn array) [58]. Laminar fluid interacts with the array to force particles or cells into a specific trajectory preset by the device. When particles or cells below the critical diameter (Dc) follow the streamline through the array gap, there is no net displacement from the original flow. When the particles exceeding the Dc move laterally in the cross-order streamline, each row moves at a predetermined angle of the micro-column offset distance [59]. Based on this principle, DLD technology can separate particles with a high-resolution ratio. Wunsch et al. showed that nano-DLD arrays can separate particles between 20 nm to 110 nm with a clear resolution ratio under the condition of diffusion and deterministic displacement competition at a low Pe number [47]. However, due to the high hydrodynamic resistance of this device, a pressure of more than 200 kPa is required to move the EV through the nanoparticle array. Hattori et al. applied an electroosmotic flow (EOF) to drive the micro-nano fluid in the chip to avoid excessive pressure; this method achieved continuous and precise separation with throughputs of 450–950 μm/s. However, the collected sample was 50−400 nm in diameter from its reservoir C, which is expected to collect exosomes. These results indicate that the purity of exosomes collected by this device needs to be improved [60] (Figure 4).

#### 3.1.3. Acoustic-Wave-Based Microfluidic Devices

**Figure 5:**
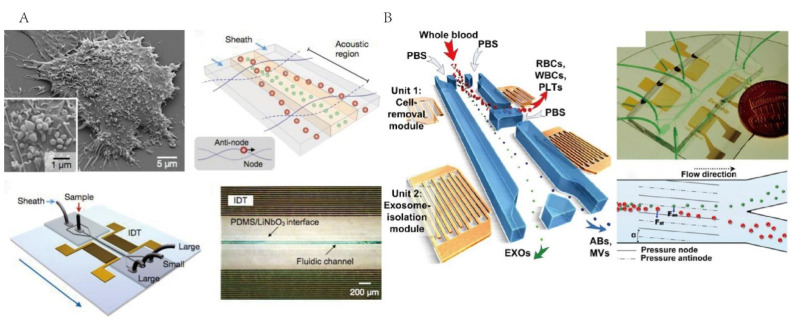
Microfluidic technology for exosome isolation based on the acoustic field. (**A**) Schematic of acoustic nano-filter for exosome isolation. Reprinted with permission [65]. (**B**) Schematic of integrated acoustic device for isolating exosomes. Reprinted with permission [48].

Acoustic microfluidic technology is a label-free exosome isolation technique by applying acoustic waves with high biocompatibility and repeatability [61,62,63]. During the separation process, the particles in the fluid are subjected to acoustic radiation forces, which are proportional to the volume of the particles. At the same time, when the particle is affected by the acoustic radiation force, it will be affected by Stokes’s law, which is proportional to the radius of the particle [64]. Therefore, larger particles are subjected to greater acoustic forces that cause them to shift their orbits further. Based on this principle, particles of different sizes can be separated. Lee et al. proposed an acoustic nano-filter system using ultrasound standing waves for the continuous filtration of microvesicles (MVs) [65]. By optimizing the design of interdigital transducers (IDT) and their underlying electronics to produce a maximal acoustic force on MVs, this type of device achieved separation yields > 90% and allowed for the isolation of exosomes from cell culture mediums and erythrocyte-derived vesicles from stored blood units (Figure 5A). Wu et al. developed a unique exosome-separation technology that integrated acoustics and microfluidics, including a cell-removal module and extracellular vesicle subgroup-separation module [48]. In the cell-removal module, larger blood components such as cell debris were removed to obtain 110 nm particles. In the next module, exosomes could then be obtained from the extra-cellular vesicle mixture with a recovery rate and purity of 82.4% and 98.4% (Figure 5B). This integrated system can separate exosomes from undiluted whole blood with a high purity and as few operations as possible. 

#### 3.1.4. Electrical-Field-Based Microfluidic Device

**Figure 6:**
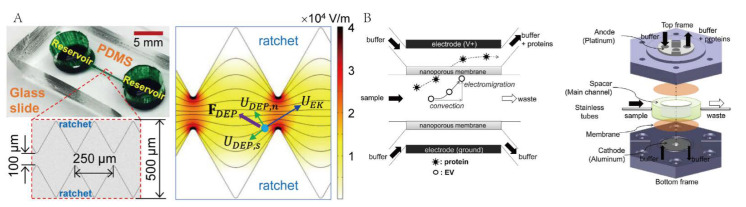
Microfluidic technology for exosome isolation based on the electrical field. (**A**) Schematic of microdevice used AC iDEP for exosomes isolation. Reprinted with permission [67]. (**B**) Schematic of isolation processes by electrophoretic migration. Reprinted with permission [55].

A variety of electrokinetic phenomena can be produced by applying AC voltage to microelectrodes placed in a solution. Among them, dielectrophoretic (DEP) and electrophoretic (EP) are mainly combined with microfluidics and used for exosome separation [49,54,55,66]. DEP describes the translational motion of neutral particles in an asymmetrical electric field due to dielectric polarization. The dielectrophoretic force is related to the particle size and the absolute permittivity. Ibsen et al. proposed an alternating-current electrokinetic (ACE) microarray chip device to isolate exosomes from undiluted plasma samples (30–50 μL) within 30 min [49]. In the isolation process, exosomes are attracted to the high-field region around the ACE microelectrode, while cells and other larger substances move to the low-field region. However, direct contact between exosomes and microelectrodes may cause adverse effects on the exosomes. Shi et al. reported a novel insulator-based dielectrophoretic (iDEP) device to rapidly isolate exosomes (within 20 min) from undiluted plasma and serum by applying a low electric field (~10 V cm^−1^) direct current (DC) [66]. This device can induce a strong non-uniform electric field (E-field) that creates a dielectrophoretic (DEP) force, which is balanced by two other electrokinetic forces, including electroosmosis (EOF) and electrophoresis (EP) near the pipette tip region, and thus it creates a trapping zone. In addition, Malekanfard et al. proposed the use of low-frequency AC electric fields to generate an oscillatory electrokinetic flow for particles focusing on a virtually “infinite” microchannel, which decreases the requirements for DC iDEP pre-focusing particles into a tight stream [67] (Figure 6A). 

EP is another technique based on the electrical properties for separating charged particles. Generally, exosomes are captured using EP to drive particles through a nanomembrane. As mentioned in Section 3.1.1, Cho et al. proposed to separate exosomes by using an electric field migration [55]. An electric field was applied to the dialysis membrane with a pore size of 30 nm. Under the action of the electric field, proteins would migrate through the membrane to the outside, and exosomes would be trapped on the membrane surface to achieve separation and enrichment (Figure 6B).

#### 3.1.5. Viscoelastic Flow-Based Microfluidic Devices

Viscoelastic flow sorting is another label-free approach for exosome separation [68]. The separation is realized using the different elastic lift of particles with different particle sizes in the solution [69,70]. Generally, exosomes do not receive enough viscoelasticity because of their small size. 

**Figure 7:**
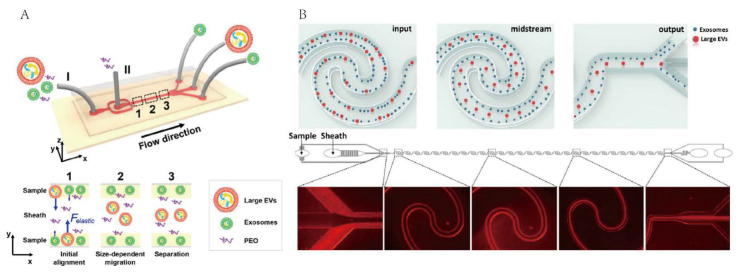
Microfluidic technology for exosome isolation based on Viscoelastic flow. (**A**) Schematic of viscoelasticity based microfluidic system. Reprinted with permission [71]. (**B**) Schematic of exosome sorting technique based on elasto-inertial. Reprinted with permission [50].

Liu et al. developed a microfluidic system for separation of exosomes from other larger EVs in cell culture mediums or serums [71]. This microfluidic chip consists of two inlets and three outlets. Samples and polyoxyethylene polymers (PEO) are, respectively, introduced into inlet Ⅰ and inlet Ⅱ; the viscoelasticity applied to the exosomes was increased by adding a small amount of biocompatible PEO to the medium (Figure 7A). As a result, larger particles were collected at the middle outlet, while exosomes were collected at the two side outlets. This method does not need complex or time-consuming operations and can finally achieve high separation purity (>90%) and high recovery (80%) of exosomes. Additionally, Zhou et al. showed a novel reverse wavy channel structure using viscoelastic flow for sorting of exosomes [50]. The structure can generate periodically reversed Dean secondary flow, which facilitates particle focusing compared with traditional straight channels (Figure 7B). The exosome’s purity and recovery were obtained as 92% and 81%, respectively.

### 3.2. Immunoaffinity Based Microfluidic

The immunoaffinity-based isolation method is based on the specific interactions of antigens and antibodies [72,73]. Generally, antibodies that capture exosomes are usually immobilized on the surface of microfluidic devices or magnetic beads [51,52,74,75]. In addition, aptamers are small oligonucleotide sequences or short polypeptides, which have been widely used as molecular probes [76]. Recently, there have been some studies that have shown that aptamers can be used to capture exosomes [53,76,77,78]. The most common biomarkers that exist on exosomes are tetraspanins, which mainly including CD9, CD63, CD81, and CD147 [79,80].

**Figure 8:**
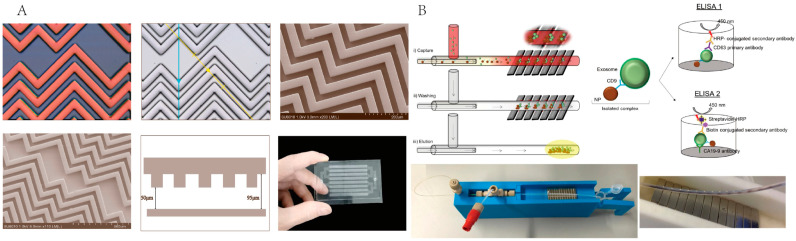
Microfluidic technology for exosome isolation based on immunoaffinity. (**A**) Schematic of the structure of ^HB^EXO-Chip for exosomes isolation. Reprinted with permission [51]. (**B**) Schematic of microdevice used magnetic nanoparticles functionalized with antibodies for exosomes isolation. Reprinted with permission [53].

Kanwar et al. developed a simple, low-cost microfluidic device (ExoChip) that can be fabricated in polydimethylsiloxane (PDMS) and functionalized with antibodies against CD63 for exosome isolation, quantification, and characterization [81]. Ten ExoChip experiments were carried out on serum obtained from five patients with pancreatic cancer and five healthy individuals, respectively. The results showed that the amounts of exosomes captured by the cancer patients were more than twice that of the healthy individuals. In order to prevent the interference of other non-specific exosomes and make the detection result more accurate, Zhang et al. developed a microfluidic device ^HB^EXO-Chip to isolate exosomes of the pancreatic cancer target Glypican-1 (GPC1) antibody [51]. The expression of GPC1 in pancreatic cancer is very high, and it is also involved in the proliferation and the metastasis of tumor cells [82,83]. It was selected as a target for exosome capture. Moreover, the microfluidic device employed a plurality of herringbone micromixers, which are beneficial for increasing the binding of exosomes to specific antibodies (Figure 8A). As a result, the device has 75% efficiency in capturing tumor-derived exosomes from plasma, as well as four-fold increase in exosomes enrichment ratios when compared to conventional methods due to its unique herringbone structure. 

Another common immunoaffinity-based method is using magnetic bead binding antibodies to capture exosomes. Sancho-Albero et al. designed a microfluidic device to isolate exosomes from whole blood using magnetic nanoparticles (Fe_3_O_4_NPs) functionalized with anti-CD63 [52] (Figure 8B). Finally, approximately 2 × 10^10^ exosomes were captured from PBS, FBS, and the whole blood in 500 μL. The use of antibodies can effectively capture high-purity exosomes. However, due to the expensiveness of antibodies, and the difficulty of separating antibodies and exosomes for the downstream application, Song et al. employed aptamers to target exosomes CD63 [53]. They developed a magnetic bead-based system using a CD63-1 aptamer for isolating exosomes from a cell culture medium. As a result, over 8.37 × 10^8^/mL exosomes were collected with over 72% purity.

## 4. Integrated Microfluidic Techniques for Exosome Separation and Analysis

Integrated microfluidic techniques can reduce off-chip operations and save time, which is beneficial to meet the needs of the rapid detection of exosomes in a large number of clinical samples. Generally, integrated microfluidic techniques consist of two compartments, including a separation zone and a detection zone. In this section, we will introduce several common detection techniques.

### 4.1. Fluorescence Detection

Fluorescence occurs when a substance is irradiated by a certain wavelength of light, and it emits light with a wavelength longer than the incident light for a short time. Fluorescence detection is the most widely used detection technology. A common approach is to use fluorescent antibodies to bind to the captured exosomes, and then characterize the exosomes using fluorescence microscopy [84,85]. Lu et al. presented an integrated microfluidic device termed ‘EXID system’ for the isolation and analysis of exosomes of the PD-L1 expression [86]. The device consists of two components: the first part is for exosome isolation and labelling, the magnetic beads were combined with anti-CD63 in the serpentine-shaped channels, this structure can lengthen the flow path to stabilize liquid flow and avoid bead clogging. The second part is used for the analysis of PD-L1 protein. Exosomes labelled with a PD-L1 fluorescence probe are captured by micropillar arrays. The result showed that 7 cell lines and 16 clinical samples were profiled with verified expression levels of PD-L1. The system can isolate exosomes and quantify biomarkers in less than 2 h, indicating a great potential for auxiliary diagnosis and individual therapy. Yu et al. proposed a highly integrated microfluidic (ExoSD) chip for exosome isolation and detection [74]. Similarly, this chip primarily incorporates two compartments: a separation zone and a detection zone. In the separation zone, a nickel (Ni) comb-like structure was designed to enhance the magnetic flux density and the magnetic field gradient acting on the immunomagnetic nanoparticles (IMNPs). IMNPs prepared by anti-CD63 can specifically capture exosomes in the cell culture supernatant. Then, in the detection zone, the fluorescent antibodies were injected in a proper order to combine to the surface antigen of the exosomes. Lastly, the cancer-cell-derived exosomes can be detected with the fluorescence signal in real time. In order to avoid sample preprocessing and solve the problem of inaccurate disease diagnosis only based on quantitative exosome concentration, Zhou et al. designed a microfluidic device termed a plasma separation and EV detection (PS-ED) chip [87]. The device included two modules: a PS module and an ED module. The PS module is composed of six looped microchannels, this design uses inertial force to quickly separate plasma and reduce mechanical damage to blood cells. The ED module is composed of four S shaped channels for exosome quantification and protein analysis based on the ELISA assay. As a result, the cancer type can be accurately confirmed and verified by clinical blood sample analysis.

### 4.2. Colorimetric Detection

The colorimetric method reflects the content of exosomes by judging the color change and the depth of the solution [88]. This method has the characteristic of a simple operation. It has a great potential in point-of-care testing (POCT) and clinical examination [89,90,91]. Chen et al. designed an integrated microfluidic system that is divided into three steps to separate and quantify exosomes from whole blood [92]. In the third step, captured exosomes were precisely quantified in an exosome quantification module, integrating multiple microchambers, micropumps, and microvalves via an enzyme-linked immunosorbent assay (ELISA), with a tyramide signal amplification technique (TSA) to greatly enhance the exosome-associated signals.

### 4.3. Surface Plasmon Resonance (SPR) Detection

SPR is a sensitive surface-analysis technique that detects changes in permittivity caused by molecules adsorbing onto heavy metal films [93,94,95]. Microfluidic devices based on SPR detection usually use light to shoot into the chip with a fixed incident angle. When the antibody on the chip captures exosomes, the refractive index will change, and the reflection intensity will change. Zhu et al. used surface plasmon resonance imaging (SPRi) combined with microfluidic chip used antibodies to capture exosomes from cell culture supernatants (CCS) [94]. Wang et al. reported an aptamer-based SPR sensor for exosome detection [96]. First, the Au film functionalized by captured DNA was used to capture exosomes, and then the target exosomes were detected, and signals were amplified by the aptamer/T_30_ linked AuNPs. Finally, the aptamer/T_30_ linked AuNPs could capture A_30_ coated AuNPs, and the target exosomes could be detected by the SPR, and their signal amplified by the double AuNP.

### 4.4. Electrochemical Detection

Electrochemical detection is a low-cost and high-sensitivity detection method. During the detection process, antibodies or aptamers are modified on the electrode, and, when exosomes specifically bind to them, electrical signals such as voltage, current, and resistance will change [77,97,98,99]. Jeong et al. designed an integrated chip termed iMEX (integrated magnetic-electrochemical exosome) for rapid exosome analyses [100]. After exosomes were captured and labelled by magnetic beads, they were detected by electrochemical sensing. The device has eight detection channels to detect exosomes sensitively from 10 μL of samples. Kashefi-Kheyrabadi et al. developed a detachable microfluidic device using an electrochemical aptasensor (DeMEA) for highly sensitive, in situ quantification of cancerous exosomes [101]. The DeMEA works by adding nanocomposite to the surface of electrodes, then aptamers against an epithelial cell adhesion molecule were fixed on the electrode surface to detect exosomes. The result indicated that the limit of detection (LOD) was 17 exosomes/μL. In addition, the detachable structure can provide an opportunity for exosome downstream analysis.

## 5. Conclusions

With the improvement in exosome research, it is recognized that exosomes are closely related to many physiological activities and the occurrence and development of diseases. Therefore, exosome isolation and analysis are very important for exosome-related research and application. However, there are many limitations in traditional methods of exosome isolation, such as expensive instruments and reagents and the consumption of a considerable amount of time. Compared with traditional methods, microfluidic technology has the advantages of high efficiency and high sensitivity for exosome separation. In recent years, with the rapid development of technology and further research, microfluidic technology has achieved efficient separation, enrichment, and multi-information detection of exosomes integrated into a single chip. This indicates that microfluidic chips have great potential in clinical research, such as point-of-care testing.

In this review, in addition to introducing the traditional exosome-separation methods, we also summarized the exosome-separation methods based on microfluidic technology as well as their pros and cons. The immunoaffinity-based microfluidic method can isolate exosomes with high purity. However, due to the high cost of antibodies, and the isolation process making it easy to cause phenotypic changes to exosomes, which is not conducive to downstream analysis, the microfluidic methods based on the physical characteristics of exosomes may be a better choice. In addition, due to the small size of exosomes and some particles being similar to their particle size, a single separation method is insufficient. In conclusion, how to balance the relationship between the purity, recovery, and flux of exosomes on microfluidic chips is a key issue. With the development of precision manufacturing technology, a variety of exosome-separation methods integrated on a chip and the integration of exosome separation and detection are perhaps helpful in solving this problem.

## Figures and Tables

**Table 1:** Conventional isolation method for exosomes.

Isolation Method	Working Principle	Advantages	Disadvantages
Centrifugation	Based on the size and density of particles	Low costs and high purity	Time-consuming and requires expensive instruments
Ultrafiltration	Based on the size of particles and the pore size of the nanomembrane	Simple operation and fast procedure	Membrane clogging easily
Size-Exclusion Chromatography	Based on the size of particles and the pore size of porous materials	Minimal damage of exosomes and high purity	Time-consuming and low efficiency
Polymer-Based Precipitation	Based on the altering the solubility of exosomes	High efficiency	Low purity

**Table 2:** Microfluidic isolation methods for exosomes.

Method	Advantages	Disadvantages	Reference	Recovery/Yield	Purity	Time Consuming
Filtration	High yield	Time consuming	ExoTIC [45]	Yield: 4~1000 fold higher than UC	NA	<3 h
	Good at isolating large samples	Complicated manufacturing process	Ciliated micropillars [46]	NA	NA	NA
DLD	High resolution	Complicated manufacturing process	Nano-DLD arrays [47]	NA	NA	NA
Acoustic microfluidic	High purity Quick separationBiocompatibility	Complicated manufacturing process	The acoustofluidic platform [48]	82%	98%	20 min
Dielectrophoretic	Quick separation	The electric field may affect the properties of exosomes	ACE microarray chip [49]	NA	NA	<30 min
Viscoelastic flow	High purity	With extra regents	Wavy microchannel structures [50]	>81%	>92%	NA
Immunoaffinity based isolation method	High specificity and purity	the expensiveness of antibodies	^HB^EXO-chip [51]	75%	NA	20 min
			Magnetic nanoparticles [52]	NA	NA	NA
			Aptamer-based exosome isolation [53]	NA	>72%	NA

## Data Availability

Not applicable.
